# Supplementary material for: Gradients in signal complexity of sleep-wake intracerebral EEG
Source: PLoS One. 2025 Mar 31;20(3):e0320648. doi: 10.1371/journal.pone.0320648 (PMC11957301; doi:10.1371/journal.pone.0320648)
Supplement: S1 Table — In general, the larger numbers of Destrieux brain regions led to several single MNIA regions mapping to multiple Destrieux brain regions. Over the frontomedial brain surface the MNIA parcellation is finer than Destrieux and required subdivision of Destrieux’s paracentral lobule and superior frontal gyrus into three sub-regions each (see text). (DOCX) [file pone.0320648.s001.docx]

| **MNIA number** | **MNIA atlas name** | **Destrieux number** | **Destrieux atlas name** | **Expanded Destrieux name** |
| --- | --- | --- | --- | --- |
| **1** | Superior and middle occipital gyri | **59** | 'S_occipital_ant' | Anterior occipital sulcus and preoccipital notch (temporo-occipital incisure) |
| **1** | Superior and middle occipital gyri | **19** | 'G_occipital_middle' | Middle occipital gyrus (O2 lateral occipital gyrus) |
| **1** | Superior and middle occipital gyri | **57** | 'S_oc_middle_and_Lunatus' | Middle occipital sulcus and lunatus sulcus S |
| **1** | Superior and middle occipital gyri | **20** | 'G_occipital_sup' | Superior occipital gyrus (O1) |
| **1** | Superior and middle occipital gyri | **58** | 'S_oc_sup_and_transversal' | Superior occipital sulcus and transverse occipital sulcus |
| **2** | Inferior occipital gyrus and occipital pole | **2** | 'G_and_S_occipital_inf' | Inferior occipital gyrus (O3) and sulcus |
| **2** | Inferior occipital gyrus and occipital pole | **42** | 'Pole_occipital' | Occipital pole |
| **3** | Cuneus | **11** | 'G_cuneus' | Cuneus (O6) |
| **3** | Cuneus | **65** | 'S_parieto_occipital' | Parieto-occipital sulcus (or fissure) |
| **4** | Calcarine cortex | **44** | 'S_calcarine' | Calcarine sulcus |
| **5** | Lingual gyrus and occipital fusiform gyrus | **22** | 'G_oc-temp_med-Lingual' | Lingual gyrus ligual part of the medial occipito-temporal gyrus (O5) |
| **5** | Lingual gyrus and occipital fusiform gyrus | **61** | 'S_oc-temp_med_and_Lingual' | Medial occipito-temporal sulcus (collateral sulcus) and lingual sulcus |
| **6** | Postcentral gyrus (including medial segment) | **46** | 'S_cingul-Marginalis' | Marginal branch (or part) of the cingulate sulcus |
| **6** | Postcentral gyrus (including medial segment) | **28/3b** | 'G_postcentral' | Postcentral gyrus + paracentral lobule (posterior) |
| **6** | Postcentral gyrus (including medial segment) | **67** | 'S_postcentral' | Postcentral sulcus |
| **7** | Superior parietal lobule | **56** | 'S_intrapariet_and_P_trans' | Intraparietal sulcus (interparietal sulcus) and transverse parietal sulci |
| **7** | Superior parietal lobule | **27** | 'G_parietal_sup' | Superior parietal lobule (lateral part of P1) |
| **8** | Parietal operculum | **41** | 'Lat_Fis-post' | Posterior ramus (or segment) of the lateral sulcus (or fissure) |
| **9** | Supramarginal gyrus | **26** | 'G_pariet_inf-Supramar' | Supramarginal gyrus |
| **10** | Angular gyrus | **25** | 'G_pariet_inf-Angular' | Angular gyrus |
| **10** | Angular gyrus | **55** | 'S_interm_prim-Jensen' | Sulcus intermedius primus (of Jensen) |
| **11** | Precuneus | **30** | 'G_precuneus' | Precuneus (medial part of P1) S |
| **11** | Precuneus | **71** | 'S_subparietal' | Subparietal sulcus |
| **12** | Posterior cingulate | **9** | 'G_cingul-Post-dorsal' | Posterior-dorsal part of the cingulate gyrus (dPCC) |
| **12** | Posterior cingulate | **10** | 'G_cingul-Post-ventral' | Posterior-ventral part of the cingulate gyrus (vPCC isthmus of the cingulate gyrus) |
| **13** | Anterior insula | **47** | 'S_circular_insula_ant' | Anterior segment of the circular sulcus of the insula |
| **13** | Anterior insula | **18** | 'G_insular_short' | Short insular gyri |
| **13** | Anterior insula | **49** | 'S_circular_insula_sup' | Superior segment of the circular sulcus of the insula |
| **14** | Posterior insula | **48** | 'S_circular_insula_inf' | Inferior segment of the circular sulcus of the insula |
| **14** | Posterior insula | **17** | 'G_Ins_lg_and_S_cent_ins' | Long insular gyrus and central sulcus of the insula |
| **15** | Gyrus rectus and orbital gyri | **62** | 'S_orbital_lateral' | Long insular gyrus and central sulcus of the insula |
| **15** | Gyrus rectus and orbital gyri | **63** | 'S_orbital_med-olfact' | Lateral orbital sulcus |
| **15** | Gyrus rectus and orbital gyri | **24** | 'G_orbital' | Medial orbital sulcus (olfactory sulcus) |
| **15** | Gyrus rectus and orbital gyri | **64** | 'S_orbital-H_Shaped' | Orbital gyri |
| **15** | Gyrus rectus and orbital gyri | **31** | 'G_rectus' | Orbital sulci (H-shaped sulci) |
| **15** | Gyrus rectus and orbital gyri | **32** | 'G_subcallosal' | Straight gyrus, gyrus rectus |
| **15** | Gyrus rectus and orbital gyri | **70** | 'S_suborbital' | Subcallosal area, subcallosal gyrus |
| **16** | Anterior cingulate | **6** | 'G_and_S_cingul-Ant' | Suborbital sulcus (sulcus rostrales supraorbital sulcus) |
| **16** | Anterior cingulate | **7** | 'G_and_S_cingul-Mid-Ant' | Anterior part of the cingulate gyrus and sulcus (ACC) |
| **17** | Middle cingulate | **8** | 'G_and_S_cingul-Mid-Post' | Middle-anterior part of the cingulate gyrus and sulcus (aMCC) |
| **17** | Middle cingulate | **66** | 'S_pericallosal' | Middle-posterior part of the cingulate gyrus and sulcus (pMCC) |
| **18** | Supplementary motor cortex | **16c** | 'G_front_sup' | Pericallosal sulcus (S of corpus callosum) |
| **19** | Medial frontal cortex | **16b** | 'G_front_sup' | Superior frontal gyrus (F1) - posterior |
| **20** | Central operculum | **4** | 'G_and_S_subcentral' | Superior frontal gyrus (F1) - middle |
| **21** | Frontal operculum | **39** | 'Lat_Fis-ant-Horizont' | Subcentral gyrus (central operculum) and sulci |
| **21** | Frontal operculum | **40** | 'Lat_Fis-ant-Vertical' | Horizontal ramus of the anterior segment of the lateral sulcus (or fissure) |
| **22** | Opercular part of the inferior frontal gyrus | **12** | 'G_front_inf-Opercular' | Vertical ramus of the anterior segment of the lateral sulcus (or fissure) |
| **23** | Triangular part of the inferior frontal gyrus | **14** | 'G_front_inf-Triangul' | Opercular part of the inferior frontal gyrus |
| **24** | Orbital part of the inferior frontal gyrus | **13** | 'G_front_inf-Orbital' | Triangular part of the inferior frontal gyrus |
| **25** | Middle frontal gyrus | **52** | 'S_front_inf' | Orbital part of the inferior frontal gyrus |
| **25** | Middle frontal gyrus | **15** | 'G_front_middle' | Inferior frontal sulcus |
| **25** | Middle frontal gyrus | **53** | 'S_front_middle' | Middle frontal gyrus (F2) |
| **26** | Superior frontal gyrus and frontal pole | **1** | 'G_and_S_frontomargin' | Middle frontal sulcus |
| **26** | Superior frontal gyrus and frontal pole | **54** | 'S_front_sup' | Fronto-marginal gyrus (of Wernicke) and sulcus |
| **26** | Superior frontal gyrus and frontal pole | **5** | 'G_and_S_transv_frontopol' | Superior frontal sulcus |
| **27** | Medial segment of superior frontal gyrus | **16a** | 'G_front_sup' | Transverse frontopolar gyri and sulci |
| **28** | Medial segment of precentral gyrus | **3a** | 'G_and_S_paracentral' | Superior frontal gyrus (F1) - anterior |
| **29** | Precentral gyrus | **45** | 'S_central' | Paracentral lobule and sulcus (anterior) |
| **29** | Precentral gyrus | **68** | 'S_precentral-inf-part' | Central sulcus (Rolando's fissure) |
| **29** | Precentral gyrus | **29** | 'G_precentral' | Inferior part of the precentral sulcus |
| **29** | Precentral gyrus | **69** | 'S_precentral-sup-part' | Precentral gyrus |
| **30** | Superior temporal gyrus | **34** | 'G_temp_sup-Lateral' | Superior part of the precentral sulcus |
| **30** | Superior temporal gyrus | **73** | 'S_temporal_sup' | Lateral aspect of the superior temporal gyrus |
| **30** | Superior temporal gyrus | **74** | 'S_temporal_transverse' | Superior temporal sulcus (parallel sulcus) |
| **31** | Middle temporal gyrus | **72** | 'S_temporal_inf' | Transverse temporal sulcus |
| **31** | Middle temporal gyrus | **38** | 'G_temporal_middle' | Inferior temporal sulcus |
| **32** | Inferior temporal gyrus | **37** | 'G_temporal_inf' | Middle temporal gyrus (T2) |
| **32** | Inferior temporal gyrus | **60** | 'S_oc-temp_lat' | Lateral occipito-temporal sulcus |
| **33** | Temporal pole and planum polare | **35** | 'G_temp_sup-Plan_polar' | Planum polare of the superior temporal gyrus |
| **33** | Temporal pole and planum polare | **43** | 'Pole_temporal' | Temporal pole |
| **34** | Transverse temporal gyrus | **33** | 'G_temp_sup-G_T_transv' | Anterior transverse temporal gyrus (of Heschl) |
| **35** | Planum temporale | **36** | 'G_temp_sup-Plan_tempo' | Planum temporale or temporal plane of the superior temporal gyrus |
| **36** | Fusiform and parahippocampal gyri | **50** | 'S_collat_transv_ant' | Anterior transverse collateral sulcus |
| **36** | Fusiform and parahippocampal gyri | **21** | 'G_oc-temp_lat-fusifor' | Lateral occipito-temporal gyrus (fusiform gyrus O4-T4) |
| **36** | Fusiform and parahippocampal gyri | **23** | 'G_oc-temp_med-Parahip' | Parahippocampal gyrus, parahippocampal part of the medial occipito-temporal gyrus(T5) |
| **36** | Fusiform and parahippocampal gyri | **51** | 'S_collat_transv_post' | Posterior transverse collateral sulcus |
| **37** | Hippocampus | **NA** | NA | NA |
| **38** | Amygdala | **NA** | NA | NA |
