# Supplementary material for: Gradients in signal complexity of sleep-wake intracerebral EEG
Source: PLoS One. 2025 Mar 31;20(3):e0320648. doi: 10.1371/journal.pone.0320648 (PMC11957301; doi:10.1371/journal.pone.0320648)
Supplement: Attachment — Submitted filename: ResponseToReviewers_auresp_2.docx [file pone.0320648.s003.docx]

RESPONSE TO REVIEWER

We thank the reviewer further comments on our manuscript. The concerns raised are reproduced in *italicized* font below, and our response follows immediately beneath.

*The authors made a substantial effort to address comments and criticism by reviewers, and I think that the manuscript is improved.*

Response: Thank you.

*Point 2 by reviewer #2 (Also raised by reviewer #3). Authors should introduce in the main text the reasoning and literature behind their choice. It is not needed that figures for review are introduced in the manuscript, but the rationale should.*

Response: In addition to explanatory text in the Discussion in our prior revision, we have now added one more citation and some edits. The second paragraph in the Discussion section describes the rationale for our choice of SE as a metric of signal complexity and the justification for our choice of parameters.

*I think that the statistical analysis should be extended to the spatial patterns, as suggested by reviewer #2*.

Response: Our prior statistical analysis was restricted to signed rank-sum comparison of each of the three sleep states (N2, N3 and REM) to the wake state (W). We accept that extension of statistical analysis to spatial considerations will strengthen the manuscript. We have now done so. The methods are a prior reviewer’s suggestion (for which we give our thanks) of using shuffled distributions within a single condition, observing cosine similarities of the shuffled variants, and comparing these across the sleep-wake states. This analysis is mentioned in the Methods, and the results appear under Results.

Finally, we also took the opportunity to make some additional minor stylistic edits to the manuscript.
