# Supplementary material for: Gradients in signal complexity of sleep-wake intracerebral EEG
Source: PLoS One. 2025 Mar 31;20(3):e0320648. doi: 10.1371/journal.pone.0320648 (PMC11957301; doi:10.1371/journal.pone.0320648)
Supplement: Attachment — Submitted filename: ResponseToReviewers.docx [file pone.0320648.s002.docx]

RESPONSE TO REVIEWERS

We thank the reviewers for commentary on our manuscript. The concerns raised are reproduced in *italicized* font below, and our response follows immediately beneath.

REVIEWER #1

*The paper presents a comprehensive and insightful analysis of Sleep-Wake Intracerebral EEG, demonstrating a strong grasp of both the theoretical and practical aspects of EEG analysis. The idea is robust, and the results are clearly articulated, offering valuable contributions to the field of neuroscience. The discussion effectively contextualizes the findings within existing literature, highlighting the paper's significance and potential impact on future research.*

Response: Thank you.

REVIEWER #2

*1. First, why was SE chosen as a neural readout? As far as I know, many different types of neural measurements can reflect signal complexity, and several were also mentioned by the authors in the manuscript (e.g., lines 247 to 249). The reason for preferring SE over others should be made clearer. A comparison between the spatial patterns reflected by SE and those reflected by other readouts is necessary to demonstrate the distinctiveness of SE.*

Response: We agree that several methods of quantifying signal irregularity are applicable to biomedical datasets. Preliminarily, we mention that simple moment-based statistics (signal standard deviation or RMS power) that only measure amplitude deviation from the mean signal are insensitive to finer variations, and related spectral methods (e.g. autocorrelation and signal bandwidth) may not be sensitive enough to diagnose subtle irregularities (Pincus, 2006). Nonlinear techniques such as Lyapunov exponents, correlation dimension and Kolmogorov-Sinai (K-S) entropy were designed for deterministic dynamical systems, and in practice require large amounts of data for their computation. Applying these methods to short times series with an unknown mix of deterministic and stochastic components – such as our data - would be misleading (Pincus, 2006). As the reviewer points out, in addition to K-S entropy, we also mention approximate entropy (AE) in the original manuscript. In fact, AE was introduced to address exactly the requirements for a metric that was model-free and applicable to short noisy datasets (Pincus, 1995). Sample entropy was subsequently introduced as a refinement to AE, to correct some of the bias inherent in AE (Richman & Moorman, 2000). Since then, SE has been used in hundreds of studies involving diverse biomedical datasets. Thus, we chose SE. We are aware of a further refinement of SE – fuzzy entropy (FE) – that is claimed to offer superior results still (Chen et at, 2009; Lau et al, 2022). However, the literature on FE for biomedical datasets is currently scant, and we chose to go with the better-established SE. (ii) A different reason for choosing SE was simply to achieve consistency with our prior work that involved SE (Kalamangalam & Chelaru, 2021). That work is cited in our manuscript, and our current results can therefore be related to it. Specifically, as we mention the manuscript, the SE maximum we observed in our prior work over the frontal lobe was confirmed to be a global (whole brain) maximum in our current work. We believe this to be a noteworthy finding.

2. *Additionally, the parameters (e.g., m and r) used in the estimations require further clarification. While following convention (line 138) is a good approach in many cases, an explicit rationale for doing so should be specified. For instance, is it possible that the spatial pattern would still occur even if a different set of parameters were selected?*

Response: Thank you for raising this issue that we glossed over in the manuscript. We have now added some text in the manuscript that discusses the choice of parameters. Referring to the definition of SE, the parameter $m$ represents the window of time that is used as a comparator. $m = 2$ represents a narrow window (just 3 points long) and therefore picks up fine time-dependent variations in the signal. Having $m$ larger elongates the time window and coarsens resolution. The parameter $r$ is the fractional standard deviation (thus, its values are constrained $0 < r < 1$). Values approaching unity would allow for large variations in successive windows and be insensitive to finer variations, and values approaching zero would be oversensitive and pick up the noise floor. Finally, combinations of small $r$ and large $m$ do not yield computable values of SE. Thus, $m = 2, r = 0.2$ are optimal choices, and have been ratified in multiple studies (Pincus, 2006). The figures below illustrate the performance of SE on our data with various parameter choice for the information of the reviewer, but do not appear in the revised manuscript.

Example SE calculations for varying choice of $r$ and $m$ parameters. Increasing $r$values (top to bottom: 0.1, 0.2, 0.5) for given $m$ yield lower values of SE and smaller differences between the states. Increasing values of $m$ (left to right: 2, 5)for a given $r$ also reduce interstate differences, and particular combinations of small $r$ and large $m$ yield uncomputable SE (top right). Thus, $r = 0.2, m = 2$ (middle left) are optimal. No combination of values however changes the overall appearances of the states relative to each other. Thus the conclusions of the manuscript remain largely robust to any interpretable combination of parameter choice.

3. *…the conclusions were drawn based on observations, but statistical analysis is needed to support them. To eliminate the possibility that the identified spatial patterns were a random effect, permutations or similarity measures could be used to demonstrate the stability of the effect. For instance, one could shuffle the SE values within a condition (e.g., shuffle SE in space) several times, and then use vectorized similarity measures (e.g., cosine similarity) to estimate the spatial dissimilarity of the randomizations across conditions to see whether the real spatial effect surpassed a threshold. Alternatively, representational similarity analysis (RSA) could be used to directly compare the cortical distribution among conditions.*

Response: The lack of statistics was a significant omission in the original manuscript. Statistical testing now been included (see additional text in Methods and Results sections).

4. …*the article is somewhat brief, and the dataset appears under-analyzed compared to standard practice, especially when compared to studies using direct cortical recordings. The brevity and inadequate analyses make it difficult to draw strong conclusions, either theoretically or from a data-driven perspective. For example, it is hard to determine whether the spatial effect was driven by the different arousal states or the chosen neural measurements. It is possible that the spatial patterns would have disappeared if different parameters or neural measurements had been used.*

Response: Our goal in this work was modest – to uncover spatial features of the distribution of signal complexity and relate the findings to the cortical spatial gradient literature - and served to extend our prior published work on this topic. The data, being derived from a public database represent normative ‘global average’ behavior (to the extent that normative data can be extracted from an epilepsy population) but are also limited in what may be achieved in intensively investigating any individual or brain area. Thus, we believe that our conclusions are robust, though necessarily limited in scope. Regarding the reviewer’s specific concerns, we have answered the question posed about the choice of neural metric and parameters above. The sleep-wake differences we demonstrate are genuine and not an artifact of metric choice. Our prior work (Kalamangalam & Chelaru, 2021) worked with a completely different dataset and arrived at similar results. The current manuscript also cites similar reports (Armonaite, et al 2023; Olejarczyk et al, 2022) that use the same MNIA dataset and employ other complexity metrics that demonstrate substantially similar findings to ours.

5. *… the figures shown in the manuscript lack a scale indicator, i.e., the correspondence between the colors in the figures and the numbers reflecting the degree of activation. Although we can infer, empirically, that the colors from blue to red reflect a range from low (0) to high (1) in response levels, an explicit colormap is necessary.*

Response: Apologies for the omission and the figures now include a color bar.

6. *… the writing is somewhat repetitive across sections. For instance, the end of the methods section (from line 145 onward) and the main results (e.g., Figures 1 and 2) describe similar results using different approaches. Instead of describing the spatial effects (i.e., where the activations occurred), more space should be dedicated to discussing the implications of the results and their association with previous studies in a logical order.*

Response: We agree there is overlap between Methods and Results. In the revised manuscript we have re-organized and edited the text to minimize such overlap. As mentioned above, our work was largely observational, and we believe we have identified patterns in signal complexity dependent on brain location and brain state in a robust manner. The implications relate to spatial variation in other structural metrics (cortical thickness, myelin content, neuronal density) that we have discussed, as well to other signal complexity metrics. One omission is discussion of the literature on the influence of sleep-wake state on information processing (and therefore spatial gradients and cortical hierarchies). We have now cited some of this latter literature and included an additional figure (Figure 3) in the context of our findings. We trust that these changes, together with the reorganization of the text and expansion of the Discussion, now have the reviewer’s approval.

REVIEWER #3

1. *None of the observations are supported by any statistical testing. No p-values and sample sizes have been reported. Because of this, the conclusions feel largely speculative. Supporting the conclusions with proper statistical validation would make the findings more convincing.*

Response: Thank you for comment, and we have addressed this in response #3 to Reviewer #2 above.

2. *SE is one way to measure signal complexity, and its definition does not allow much insights into the neural dynamics. Additionally, SE is sensitive to hyperparameter selection. How would these gradients change if m and r were adjusted? Can the authors provide a rationale for setting r at 0.2?*

Response: Thank you for comment, and we have addressed this in response #2 to Reviewer #2 above.

3. *For more rigorous conclusions, it would be beneficial to explore other complexity metrics that provide more detailed insights into the nature of neural dynamics.*

Response: Thank you for comment, and we have addressed this in response #1 to Reviewer #2 above.

4. *The paper could benefit from clearer structure. Currently, key conclusions are mixed with observations, making it challenging to follow the main arguments.*

Response: Thank you for comment, and we have addressed this in response #6 to Reviewer #2 above.

5. *Lines 98-100 describe the main question of the study, but the text does not clearly provide an answer. A more direct connection between the results and the research question would improve clarity.*

Response: Lines 98-100 in the original manuscript indeed provide one specific question raised in the study. We did provide a direct answer (Line 258 of the original manuscript), but we have now rephrased the sentence in the revised manuscript to bring out the connection between the question and this specific observation, as the reviewer suggests.

6. *How were the SE values normalized across sleep stages to allow for appropriate comparisons?*

Response: Apologies for the omission. A sentence regarding the normalization method now appears in the caption to Figure 1. Essentially, mean SEs were normalized to the unit interval by defining SE_n_ = (SE – SE_min_)/(SE_max_ – SE_min_) where SE_max_ was the maximum SE over all brain areas and conditions and SE_min_ was the minimum.

7. *Minor comment: Please add color bars to all figures.*

Response: Thank you for comment, and we have addressed this in response #5 to Reviewer #2 above.

REFERENCES

Armonaite K, Nobili L, Paulon L, Balsi M, Conti L, Tecchio F. Local neurodynamics as a signature of cortical areas: new insights from sleep. Cereb Cortex. 2023;33(6):3284-92.

Kalamangalam GP & Chelaru MI (2021). Functional connectivity in dorsolateral frontal cortex: Intracranial electroencephalogram study. Brain Connect 11(10):850-64.

Lau ZJ et al (2022). Brain entropy, fractal dimensions and predictability: A review of complexity measures for EEG in healthy and neuropsychiatric populations. Eur J Neurosci 56:5047–5069.

Olejarczyk E, Gotman J, Frauscher B. Region-specific complexity of the intracranial EEG in the sleeping human brain. Sci Rep. 2022;12(1):451.

Pincus SM (1995). Approximate entropy (ApEn) as a complexity measure. Chaos 5, 110–117.

Pincus SM (2006). Approximate entropy as a measure of irregularity for

psychiatric serial metrics. Bipolar Disord 8: 430–440.

Richman JS & Moorman JR (2000). Physiological time-series analysis using approximate entropy and sample entropy. Am J Physiol Heart Circ Physiol 278(6):H2039-49.
